# Short communication: Clinical evaluation of pea sprout extract in the treatment of hair loss

**DOI:** 10.1002/ptr.6528

**Published:** 2019-11-03

**Authors:** Torsten Grothe, Franziska Wandrey, Cornelia Schuerch

**Affiliations:** ^1^ Mibelle Group Biochemistry, Mibelle AG, Bolimattstrasse 1, 5033 Buchs AG Switzerland

**Keywords:** FGF7, hair growth, hair loss, noggin, pea sprout extract

## Abstract

Hair loss affects millions of people worldwide, but currently available treatment options are often dissatisfying due to side effects or limited efficacy. Pea sprout extract has been shown to improve hair density when applied topically, but its mode of action and effectiveness upon oral administration remain unknown. Our study has now shown that the application of a fluid containing 2% pea sprout extract on a defined scalp zone of 10 volunteers enhances the expression of defined genes relevant for hair, namely fibroblast growth factor‐7 (FGF7) and noggin, by 56 and 85%, respectively. Additionally, a subsequent pilot nutrition intervention study in 21 volunteers proved that pea sprout extract is also effective when consumed as dietary supplement. The daily intake of 100 mg pea sprout extract (AnaGain™ Nu) for 8 weeks significantly reduced hair loss already after 28 days of treatment (*p* < 0.002). No adverse events were reported. Consequently, pea sprout extract may be an effective means to safely promote hair growth and reduce hair loss in individuals experiencing excessive hair shedding.

## 1 INTRODUCTION

Healthy hair serves many physiological functions and is vital to identity. It grows at a rate of 0.35 mm/day and around 100 hairs are shed daily. The hair growth cycle is characterized by three distinct phases: the phase of growth (anagen), a transitional period of apoptosis‐mediated regression (catagen) and relative quiescence (telogen), where the hair is released from the follicle and falls. Through cyclical loss and new‐hair growth, the number of hairs remains relatively constant with about 85–90% of hair follicles being anagen (Murphy & Zito, 2018). However, if the proportion of hair in the telogen phase increases and the percentage of anagen hair declines, diffuse hair loss can be observed. By age 50, about every second man and women is affected by slow, involuntary hair shedding, which can be caused by a variety of factors including hormonal changes, malnutrition, stress, medication use or family history (Rogers & Avram, 2008).

Although a number of therapies are available (e.g. finasteride), patients are often dissatisfied due to side effects of the medications and they are seeking safe, natural ingredients to attenuate hair loss (Sadick, Callender, Kircij, & Kogan, 2017).

AnaGain™ Nu, a water‐soluble extract prepared from edible organic pea sprouts (*Pisum sativum* L.), has been developed by bioassay guided product development to stimulate hair growth and reduce hair loss. Pea sprouts are a rich source of various nutrients like biotin and L‐arginine as well as secondary plant metabolites such as isoflavones, which have been suggested to promote hair growth in experimental models (Zhao, Harada, Kurihara, Nakagata, & Okajima, 2011).

A previous cosmetic study has already demonstrated that the topical application of the pea sprout extract increased hair density by improving the ratio of anagen and telogen hair (Schmid, Belser, & Zuelli, 2013). However, it is not known yet how the pea sprout extract modulates the hair cycle, and whether it is also effective when consumed orally. Therefore, the purpose of this research program was a) to analyze the effect of the pea sprout extract on gene expression in plucked hair follicles and b) to evaluate the efficacy of the extract in reducing hair loss when consumed as a food supplement.

## 2 MATERIALS AND METHODS

### 2.1 Gene expression analysis

10 volunteers (4 women and 6 men) aged 46 to 60 years were asked to apply a scalp product containing 2% of a pea sprout extract (AnaGain™, Mibelle AG, Switzerland) on a test site at the back of their head twice daily for two weeks. Before and at the end of the study, 20 hairs were plucked from the test site, pooled, cut to about 1 cm length and stored at −80°C. The expression of selected markers was analyzed using the RT‐qPCR method on mRNA extracted from different hair pools. Analysis of gene expression was performed in duplicates (*n* = 2) using a dedicated PCR array containing 32 target genes important for hair physiology. PCRs were run with the LightCycler® System (Roche Molecular System Inc.) and results were normalized to the average expression of two housekeeping genes (RPL13A, GAPDH) using the ΔCt method. In parallel two further test samples, extracts prepared from *Carlina acualis* L. and *Polygonum multiflorum* Thunb., respectively, have been investigated in 18 additional volunteers under the same method as described above.

### 2.2 Food supplement study

The pilot study enrolled 21 healthy Caucasians (22 to 63 years) with mild to moderate hair loss (≥100 lost hairs daily). Reasons for exclusion from study participation included amongst others obvious hair pathologies, severe systemic or dermatological disease, use of any topical (drug containing) or cosmetic anti‐aging product on the test areas, a food allergy against legumes and the participation in any other clinical study or the use of experimental drugs involving the test areas.

The study was performed at the Skin Test Institute, Neuchâtel, Switzerland in accordance with the ethical principles for medical research in human subjects (Declaration of Helsinki). All participants gave informed consent after study details were explained.

Subjects consumed 100 mg of a pea sprout extract (AnaGain™ Nu, provided by Mibelle AG, Switzerland) dissolved in 200–250 ml of cold liquid of their choice such as water or fruit juice once daily for eight weeks.

At baseline (t_0_), day 28 (t_1)_ and day 56 (t_2_), volunteers collected their hairs lost on the comb/brush in the morning and evening and participated in a dermatological assessment on the scalp. The collected hair was counted by a trained laboratory technician. Scalp photographs were made using Visioface® (Courage & Khazaka). At the end of intervention (t_2_), a questionnaire was filled in by volunteers to evaluate the satisfaction on treatment and the hair condition.

Participants were allowed to continue their regular hair washing routine during the study period, except for the use of products against hair loss.

### 2.3 Statistics

For all measured parameters, paired data were tested for statistical significance by non‐parametric permutation analysis using StatXact (Version 5, 2001, Cytel Software Corporation, Cambridge, MA, USA). Unless not stated differently, results are presented as means plus standard deviation.

## 3 RESULTS

### 3.1 Gene expression analysis

The application of the pea sprout extract for two weeks strongly activated the expression of fibroblast growth factor 7 (FGF7) and noggin. This was reflected by a decrease of the average ΔCt of FGF7compared to the housekeeping genes from 13.02 to 12.38, corresponding to an average increase in expression of 56%. The average ΔCt of Noggin compared to the housekeeping genes decreased from 15.44 prior to application of the pea sprout extract to 14.56 after two weeks of treatment. This corresponds to an average increase of Noggin expression of 85%. Overall, FGF7 was stimulated in 8 and noggin in 7 out of 10 participants. The two other tested plant extracts did not affect FGF7 or Noggin gene expression or any other tested target gene (data not shown).

### 3.2 Food supplement study

Of 23 volunteers (mean age 43.9 years ±12.4 years), 2 dropped out for medical or professional reasons, leaving 3 men and 18 women completing the study.

**Figure 1:**
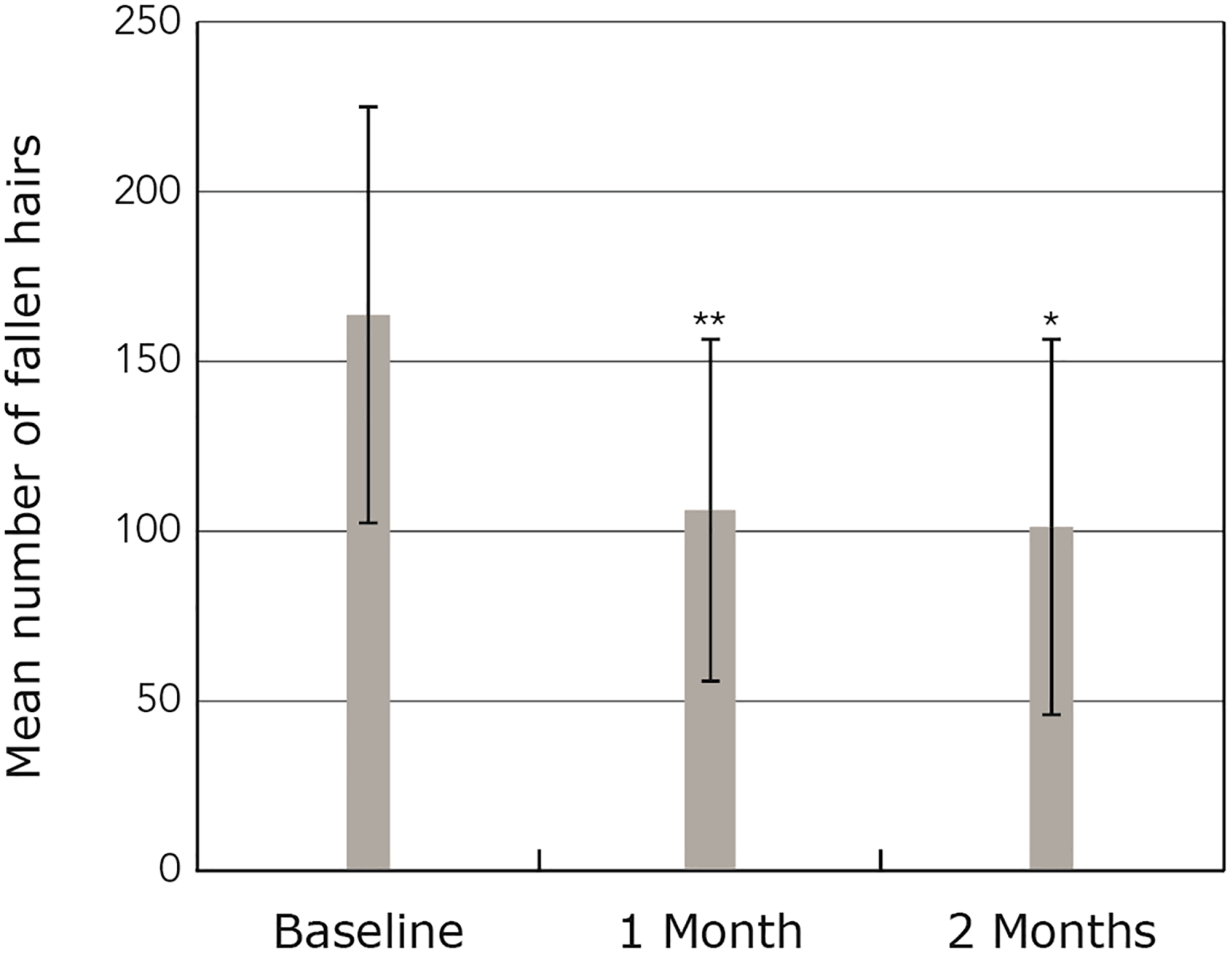
Change in the number of hairs lost per day after one and two months of supplementation with pea sprout extract; * denotes *p* = 0.0002 vs. t0, ** denotes *p* < 0.0002 vs. t0 [Colour figure can be viewed at wileyonlinelibrary.com]

After two months of treatment with the pea extract, 95% of participants experienced a reduction of hair loss. Within the first month, the mean number of hairs lost decreased by 33.9%, from 163.7 ± 61.2 at baseline to 105.7 ± 50.6 at t1 (*p* < 0.0002; Figure 1). Within the second month of supplementation, a further attenuation of hair loss was documented (t_2_: 100.4 ± 56.2, *p* = 0.0002 vs. t_0_).

The efficacy was confirmed by the subject self‐assessments. 86% of volunteers reported a reduction of hair loss after eight weeks of treatment with the pea sprout extract (*p* ≤ 0.05), 71% perceived an improvement of the overall hair condition (*p* > 0.05).

The food supplement was well tolerated without any signs of side effects.

## 4 DISCUSSION

Results of this pilot study demonstrated that the supplementation of 100 mg pea sprout extract daily safely and effectively decreased hair loss in individuals experiencing hair shedding. These findings are in line with recent evidence reporting a significant improvement of terminal hair count in men with androgenic alopecia (Nichols, Bosshardt Hughes, Cannazza, & Zaiac, 2017) and women with self‐reported hair thinning (Ablon & Kogan, 2018) upon polyphenol supplementation.

The use of anti‐oxidative and anti‐inflammatory ingredients seems reasonable for treatment of hair loss, because its multifactorial pathogenesis involves factors (e.g. stress, hormones or environmental exposure) that promote a pro‐oxidant and pro‐inflammatory environment in the follicle which may lead to a dysregulation of the hair follicle cycling. Oxidative stress stimulates the release of pro‐inflammatory cytokines such as Il‐1 and TNF‐α known to enhance apoptosis, cause follicular regression and premature termination of the anagen phase (Farris, Rogers, McMichael, & Kogan, 2017).

**Figure 2:**
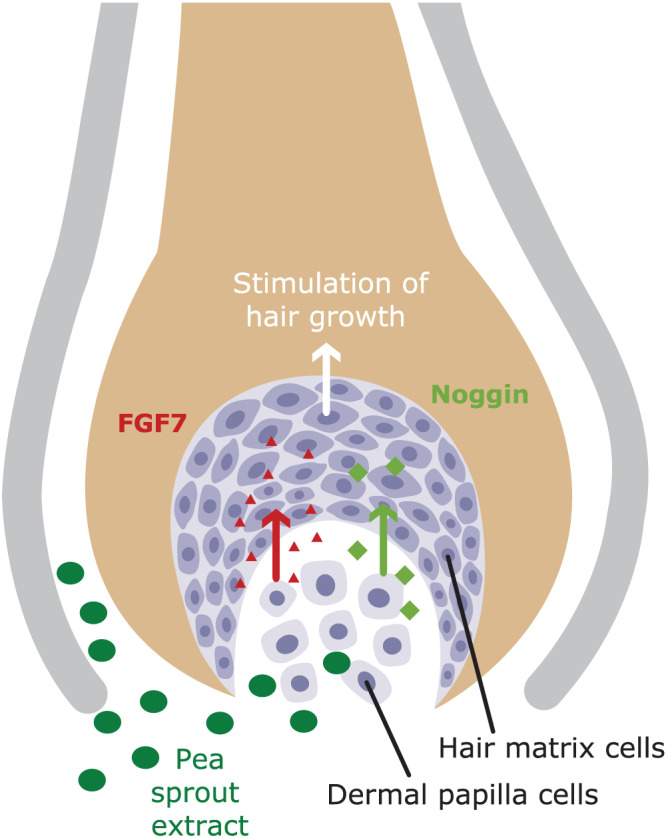
Proposed mode of action of the pea sprout extract AnaGain™ Nu [Colour figure can be viewed at wileyonlinelibrary.com]

In addition to its anti‐inflammatory and anti‐oxidant properties, pea sprout extract has been shown to have direct effects on hair growth by stimulating the expression of genes FGF7 and noggin, two well‐known signaling compounds important for the induction of a new hair growth phase (Figure 2) after two weeks treatment only. In comparison to the pilot study, changes in gene expression profile could be expected after a much shorter study duration. Therefore, a longer treatment period was chosen for the pilot study to observe changes in hair density, which are a downstream result of the gene expression changes. Additionally, the validity of the results was supported by the fact that the two other plant preparations tested in parallel did not affect gene expression at all and therefore could be considered an internal placebo control group. However, Noggin is suggested to function indirectly by inhibiting the activity of bone morphogenetic protein 4 (BMP4), a protein suppressing the telogen‐anagen transition (Plikus et al., 2008). FGF7 is a signaling factor participating in stimulating hair germ cells to proliferate and initiate a new hair cycle (Greco et al., 2009).

In conclusion, food supplements and topical solutions containing the pea sprout extract may help reduce hair loss and promote hair growth in individuals experiencing hair shedding without side effects. Placebo‐controlled interventions with a larger population size and longer study duration are desirable to confirm its effectiveness.

**Abbreviations**
AGA: androgenetic alopecia
BMP4: bone morphogenetic protein 4
FGF: fibroblast growth factor‐7
GAPDH: glyceraldehyde 3‐phosphate dehydrogenase
Il‐1: interleukin‐1
mRNA: messenger RNA
PCR: polymerase chain reaction
RPL13A: ribosomal protein 13A
TNF‐α: tumor necrosis factor alpha
TGF‐β: transforming growth factor beta

## CONFLICT OF INTEREST

No conflicts of interest were declared.
